# The utility of comparative models and the local model quality for protein crystal structure determination by Molecular Replacement

**DOI:** 10.1186/1471-2105-13-289

**Published:** 2012-11-05

**Authors:** Marcin Pawlowski, Janusz M Bujnicki

**Affiliations:** 1 Laboratory of Bioinformatics and Protein Engineering, International Institute of Molecular and Cell Biology, Trojdena 4, Warsaw, PL-02-109, Poland; 2 Laboratory of Bioinformatics, Institute of Molecular Biology and Biotechnology, Faculty of Biology, Adam Mickiewicz University, Umultowska 89, Poznan, PL-61-614, Poland

**Keywords:** Molecular replacement, MR, MQAP, Model quality assessment, Protein structure prediction

## Abstract

**Background:**

Computational models of protein structures were proved to be useful as search models in Molecular Replacement (MR), a common method to solve the phase problem faced by macromolecular crystallography. The success of MR depends on the accuracy of a search model. Unfortunately, this parameter remains unknown until the final structure of the target protein is determined. During the last few years, several Model Quality Assessment Programs (MQAPs) that predict the local accuracy of theoretical models have been developed. In this article, we analyze whether the application of MQAPs improves the utility of theoretical models in MR.

**Results:**

For our dataset of 615 search models, the real local accuracy of a model increases the MR success ratio by 101% compared to corresponding polyalanine templates. On the contrary, when local model quality is not utilized in MR, the computational models solved only 4.5% more MR searches than polyalanine templates. For the same dataset of the 615 models, a workflow combining MR with predicted local accuracy of a model found 45% more correct solution than polyalanine templates. To predict such accuracy MetaMQAPclust, a “clustering MQAP” was used.

**Conclusions:**

Using comparative models only marginally increases the MR success ratio in comparison to polyalanine structures of templates. However, the situation changes dramatically once comparative models are used together with their predicted local accuracy. A new functionality was added to the GeneSilico Fold Prediction Metaserver in order to build models that are more useful for MR searches. Additionally, we have developed a simple method, AmIgoMR (Am I good for MR?), to predict if an MR search with a template-based model for a given template is likely to find the correct solution.

## Background

In macromolecular crystallography, the determination of a 3D structure of a molecule of interest involves the gathering of the intensity and phase information for a crystal diffraction pattern, from which an electron density map can be calculated. Unfortunately, in a diffraction experiment, only the intensity is measured, and information about phases is lost. The missing information must be provided either from independent experiments or must be estimated from other sources. The Molecular Replacement (MR) technique is an approach with which to approximate the phase information from a structure of a related macromolecule or from a theoretical model of the macromolecule under investigation (called a “search model”). MR involves the placement (both the orientation and the position) of a search model in the asymmetric unit of the target crystal [1]. The placements are scored by an MR search function that measures the agreement between the theoretical and the observed structure factors [2-7]. The success of MR depends on two factors: the fraction of the asymmetric unit for which there is a suitable model, and the global model quality (e.g. RMSD after optimal superposition) of the search model. In most cases of successful MR applications for determination of protein structures, the sequence identity between the target and the template is at least 35% [8].

Traditionally, typical MR experiments involved homologous structures rather than theoretical models of the target protein. Anna Tramontano and coworkers tested the utility of theoretical models submitted to the Critical Assessment of Techniques for Protein Structure Prediction (CASP) experiment. They were able to obtain an interpretable electron density map for 28 out of 56 models [9]. In an earlier work, that group reported that GDT_TS (Global Distance Test, see Methods for explanation of this measure) was a better indicator of the model utility for MR than RMSD. They also showed that models with GDT_TS < 80 were never successful in MR, while GDT_TS >84 was sufficient to guarantee MR success [10].

It is obvious that the success of MR depends on the accuracy of the search model. Unfortunately, this parameter is unknown until the native structure of the target protein is solved. However, in the structural bioinformatics community, Model Quality Assessment Programs (MQAPs) were developed, which predict the accuracy of protein structure models (i.e. without the knowledge of the true structure), both globally and for individual residues. The existing MQAPs are based either on physical effective energy obtained from fundamental analysis of the particle forces or on empirical pseudo-energy derived from known protein structures [11]. MQAPs can be divided into two other categories: 1) “true MQAPs” - methods capable of assessing quality for *single* models, without using any alternative models (decoys) for the target protein; 2) “clustering MQAPs” – methods that rely on structural comparisons between *a number of alternative models* generated for the target sequence. According to the CASP7, CASP8, CASP9 experiments, where plenty of models based on varied prediction methods are compared, clustering approaches significantly outperform “true MQAPs”, especially when ranking models according to their accuracy. However, in cases when only one or very few alternative models are available, the gap between “clustering MQAPs” and “true MQAPs” is marginal, with “true MQAPs” performing better in extreme cases [12,13].

The aim of our work was to investigate whether protein structure prediction in combination with local model quality assessment can effectively increase the success rate of MR in comparison to the polyalanine-based MR. First, we developed an automatic MR workflow that adds information about the local model accuracy to the 3D structure of a search model. Second, we demonstrated that the knowledge of real local accuracy of a model has significant positive impact on the success rate of MR. Finally, we showed that predicted local model accuracy, according to current clustering MQAPs, could also improve the utility of computational models for MR.

## Methods

### Dataset of models

The analysis was performed on structure factors deposited in the Protein Data Bank (PDB, http://www.rcsb.org) for 11 proteins (MR targets), each of which had one molecule in the asymmetric unit (AU) and resolution ≤ 2 Å. All of these proteins had previously been targets in the CASP7 experiment and had their structure factor data deposited in the PDB. Each protein from the dataset was used to query the DALI program [14] to find structurally similar proteins (Z-score ≥ 3). Our goal was to analyze the utility of models for molecular replacement rather than to compare the utility of various search tools for finding tentative models for molecular replacement. For this reason we selected DALI rather than sequence-based homology search tools that only predict structural similarity.

Based on these DALI alignments, models of reference proteins were generated with MODELLER [15], a model-building program that minimizes the violation of stereo-chemical constraints as well as restraints derived from the template(s), and yields the canonical set of atoms for each residue. Unaligned regions (fragments corresponding to insertions in the target sequence) longer than 8 aa were removed from these models (we had tested the following thresholds of minimal length of unaligned regions to be removed: 2, 4, 6, 8, 10, >14, >18; the best results we had got for the 8 aa threshold, data not shown). As a result, 615 comparative models were created for the 11 proteins. Additional file 1: Table S1 presents the basic statistics of these models.

The average model cRMSD from the corresponding native structure was 1.76 Å, the mean GDT_TS was 73, and the average model completeness (the ratio of the number of residues in a model to the number of residues in the target sequence) was 89%. The most accurate models were created for protein 2gw2 (average GDT_TS: 92.7, RMSD: 1.03 Å), the least accurate ones for 2h58 (average GDT_TS: 58.0; RMSD: 1.94 Å).

### The temperature factor

The temperature factor or B-factor can be thought of as a measure of how much an atom oscillates or vibrates around the position specified in the model. For instance, atoms at side-chain termini exposed to the solvent are expected to show more freedom of movement than atoms in the densely packed protein core. If the temperature factor B_j_ is taken as a measure of a thermal motion of atom j, then B_j_ is related to the mean-square displacement of the atom from its average position as follows:

(1) $$\beta_{j}=8\pi^{2}\left\{u_{j^{2}}\right\}$$

where: u_j_^2^ is the mean-square displacement of the atom from its average position in all (*x,y,z*) directions.

Since B-factor relates to uncertainties in atomic positions, we decided to apply the above equation to recalculate the B-factor of an atom on the bases of its accuracy, and then use two MR programs that take into account B-factor values as the indicator of local accuracy of a model. The idea had been also proposed by Read during the CASP 7^th^ conference [16].

### Global and local accuracy of a model

In order to compute real global and local accuracy of the models in our study, the LGA [17] program was used in a sequence-dependent mode. Each model was superimposed onto its corresponding native structure and then the deviations between the atoms in the model and their counterparts in the native structure were measured.

We also used two parameters to measure the global accuracy of a model. The first one is a root mean square deviation (RMSD) between corresponding C-α atoms of the target and model structure. The second one is a Global Distance Test (GDT_TS). The latter measure corresponds to the average value of fractions of C-α atoms in the model that are placed within the distance of 1, 2, 4, or 8 Å from the corresponding C-α atoms in the native structure.

In addition, we also tested the predictions yielded by two Model Quality Assessment Programs (MQAPs): MetaMQAP and MetaMQAPclust. The MetaMQAP program [13] uses a machine learning approach to assess the deviation of C-α atoms in a model. In order to operate, MetaMQAP combines the output from a number of model quality assessment programs (MQAPs), including VERIFY3D [18], PROSA [19], BALA-SNAPP [20], ANOLEA [21], PROVE [22], TUNE [23], REFINER [24], and PROQRES [25]. In addition, the MetaMQAP prediction is based on the following residue features: secondary structure, solvent accessibility, and residue depth. The MetaMQAP method is a “true MQAP” - it is capable of producing model quality estimation based on just one single model.

MetaMQAPclust requires a set of models as an input. It functions similarly to the QMEANclust method [26]. First, it ranks all models according to the MetaMQAP score. Then, a 3D-Jury [27] procedure is executed for the 15% of top-ranked models. The 3D-Jury score of a residue *i* in a model *j* is the mean distance of the residue to corresponding residues in the pairwise superposition of the considered structures:

(2) $$S_{\mathrm{ij}}=\frac{1}{N-1}\sum_{k=1,k\neq j}^{N}d_{\mathrm{ijk}}$$

where: S_ij_ is the score of residue *i* in a model *j*. N is the number of the 15% of top-ranked models, d_*ijk*_ is the distance between *i* residues in superimposed models *j* and *k*.

In the CASP9 experiment, MetaMQAP and MetaMQAPclust were shown to belong to the groups of best performing “true MQAPs” and “clustering MQAPs”, respectively [28].

### Using local model accuracy of a model for MR

The main goal of the study was to investigate whether a combination of local model quality assessment with structure prediction can effectively increase the success rate of MR (as compared to the polyalanine-based MR). Accordingly, local model quality was used to modify the B-factor values of a search model which then in turn was used to find MR solutions using MOLREP [29] and AMoRe [30]. In our procedure, the two programs took into account the B-factor values of the atoms as indicators of uncertainties in these atoms’ positions. In addition, we also tested whether all-atom models have the same utility for MR as the backbone-atom ones. Taking all of these questions into account, we created ten groups of search models, as listed below:

(a) ALL_20 – full atom models, no information about local model accuracy was used - the B-factor of each atom was set to 20.

(b) ALL_IDEAL – B-factor values were modified (see equation 1) according to the real local accuracy of a model, calculated by superposition of the model onto the true structure.

(c) BACKBONE_20 – models as in ALL_20, but containing backbone atoms only (all side-chain atoms were removed).

(d) BACKBONE_IDEAL - models as in ALL_IDEAL, but backbone atoms only (all side-chain atoms were removed).

(e) ALL_CA_IDEAL – B-factor values of each residue in the model were modified (see equation 1) according to the real local accuracy of the position of this residue’s C-α atom.

(f) BACKBONE_CA_IDEAL - models as in ALL_CA_IDEAL, but backbone atoms only (all side-chain atoms were removed).

(g) MetaMQAP-evaluated - B-factors values were modified (see equation 1) according to the local model accuracy predicted by the MetaMQAP method [13] (see equation 2) (see below). Since MetaMQAP predicts deviation only for C-α atoms, the B-factor value predicted for each C-α atom was transferred to all main-chain atoms of the residue under consideration. In addition, all side-chain atoms were removed.

(h) MetaMQAPclust-evaluated – similar to MetaMQAP-evaluated, but the local model accuracy was predicted by the new MetaMQAPclust method instead of MetaMQAP.

(i) POLYALA – plyalanine templates - template structures converted to polyalanine models.

(j) POLYALA_20 – similar to POLYALA, but the B-factor value of each atom was set to 20.

The groups from a) to h) contain different modifications of each of the initial 615 models. Additionally, we also run MR with polyalanine templates (i-j), which we consider as the baseline regarding to the polyalanine-based MR procedure.

### Molecular Replacement and model building workflow

**Figure 1:**
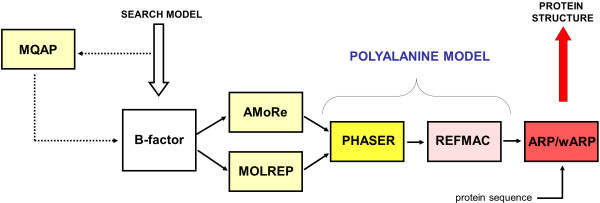
**The schema of the MR workflow utilizing local model quality.** A) The B-factor value of each atom in a search model is modified according to the corresponding deviation. B) The initial placement of a search model in the asymmetric unit is done by AMORE and MOLREP programs, then 10 best scored solutions of each of the programs are selected. C) Then, such solutions are converted to polyalanine models and given as an input to PHASER. D) Next, the top solution of the PHASER program is given as the input to Refmac5 to perform restrained refinement. E) Finally, the structure of the target is built automatically using the ARP/wARP program.

The MR procedure and model building were run in a completely automatic fashion (see Figure 1). The workflow consisted of the following steps:

A. The B-factor value of each atom was modified according to the local accuracy of a search model. In case of MetaMQAP and MetaMQAPclust, which predict only the deviations for C-α atoms, the deviations of other atoms for a given residue were assumed to be equal to the B-factor value of the C-α atom of the residue.

B. The placement of a search model in the asymmetric unit was done by two programs, MOLREP [29] and AMoRe [30], included in the CCP4 suite [31]. Both programs were executed with the default parameters (apart from a MOLREP non-default option - “use of the original model Bfactor”, which was turned on). The default resolution limit of 3 Å was used for both programs.

C. For each method (MOLREP & AMORE) the 10 best orientations of a search model in AU were collected. Then, the PHASER [32] was used to refine all of these solutions. The PHASER program was run in a refinement mode with the default parameters.

D. The best orientation according to the PHASER program was given as input to REFMAC [33] to perform 20 cycles of restrained refinement without prior phase information.

E. Finally, by using the ARP/wARP program [34], the observed structure factors (experimental structure factors deposited in PDB) and reconstructed phases (theoretical data computed for the REFMAC solution) were used to build the structure of the target protein. The ARP/wARP program was executed with the default parameters.

## Results

We performed MR for 11 known protein structures with a resolution better than 2 Å and one molecule per asymmetric unit. For these proteins, 615 comparative models were created in total (see Methods for details). To investigate if the local accuracy of a search model is important for MR, we tested eight variants of the modifications of these models. In addition, as a control study, we also tested two sets of polyalanine templates (see Methods). Finally, 6150 independent MR phasing attempts were performed.

In order to count as a successful MR case, at least half of the C-α chain must have been built by our Molecular Replacement workflow ( Additional file 1: Figure S2 presents histograms of the fraction of rebuild C-α atoms). First, we checked whether the use of known or predicted local model accuracy improved the utility of theoretical models in MR. Second, we assessed which measure of the global model quality was the best indicator of a model suitability for MR. Finally, we analyzed when MR benefited most from the use of theoretical models when the information about the local accuracy of these models was given.

### The absolute real local accuracy of search models increases the likelihood of successful MR calculations

This section deals with testing if local accuracy can improve the MR success ratio. Thus, as it was described in the Methods paragraph, we tested 10 different variants of search models, which were varied in the following characteristics: the type of the local accuracy of a model (real, predicted), the subset of atoms used to define the structure of a search model (all-atom model, back-bone model), the type of a search model (homology model, polyalanine templates).

**Figure 2:**
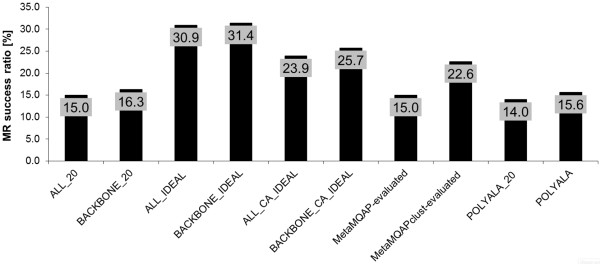
**The impact of local model quality on MR success ratio.** The success ratio is the fraction of correct MR solutions found by models of a given type. Among the types are: 1) models not utilizing local model quality:ALL_20, BACKBONE_20; 2) models modified according to ideal local quality of each atom: ALL_IDEAL, BACKBONE_IDEAL; 3) models modified according to ideal local quality of C-α atoms: ALL_CA_IDEAL, BACKBONE_CA_IDEAL; 4) models modified according to predicted local quality by MQAPs: MetaMQAP-evaluated, MetaMQAPclust-evaluated; 5) templates converted into polyalanine models.

According to the data presented in Figure 2, all-atom models without any information about local model quality (ALL_20) were effective in 15.0% of the MR cases analyzed. Similarly, backbone-atom variants of these models (BACKBONE_20) were successful in 16.3% of the cases, showing that the removal of side-chain atoms slightly increases the model utility for MR. 15.6% success ratio was achieved when polyalanine templates (POLYALA) were used. Interestingly, the use of polyalanine templates with B-factor values set to 20 (POLYALA_20) had significantly lower success ratio (14.0%), suggesting that B-factor values bring an added value to the MR procedure - the original B-factor values increased the success ratio by 11.4 % compared with the POLYALA_20 search models.

When the real local accuracy of a model was used to modify the B-factor values, all-atom models (ALL_IDEAL) and backbone-atom models (BACKBONE_IDEAL) were able to solve respectively 30.9% and 31.4% of MR cases. Search models modified only according to the real deviation of C-α atoms (with the B-factor values of the non- C-α atoms in a given reside were set to the B-factor value of the C-α atom) were less useful for MR. MR searches with ALL_CA_IDEAL models found correct solutions in 23.9% of cases, while for BACKBONE_CA_IDEAL models the success ratio was 25.7%.

In conclusion, we demonstrated that: 1) backbone models were more efficient in MR than the corresponding all-atom models; 2) the classic MR based on either homologous structures or comparative models had a success ratio of 16.3% at most (the best were the backbone-atom models with B-factor values set to 20); 3) comparative models were slightly more useful for MR than the corresponding polyalanine templates; 4) the use of real local accuracy almost doubled the utility (success in MR) of all-atom models (31.4% vs. 16.3%); 5) when only the local quality of C-α atoms was used the success ratio increased 1.58 times only (25.7% vs. 16.3%).

### The role of global model quality in predicting the model utility for MR

Global model quality is defined here as the overall deviation between the model and the reference (i.e. experimentally determined) structure. The root-mean-square deviation (RMSD) between the corresponding atoms of the model and the native structure is one of the most commonly used measures of global model quality. However, it exhibits many problems, such as strong dependence on protein length and the hypersensitivity to the presence of a few major outliers. In contrast to RMSD, GDT_TS is a measure that calculates the largest set of the C-α atoms falling within a defined distance cutoff of their position in the native structure. Therefore, GDT_TS score measures the fraction of correctly predicted structure and is less sensitive to larger errors localized in a few regions of the model. The GDT_TS score was adopted as the main assessment criterion for the Critical Assessment of Structure Prediction [35].

To analyze how efficient the RMSD and GDT_TS measures are for predicting whether a model is suitable for MR, we conducted the ROC curve analysis. For each predictor, the Area Under the ROC Curves (AUC) was measured. In such analysis a perfect prediction would yield AUC *=* 1, whereas AUC *=* ½ would suggest a random prediction. Among predictors, not only did we test RMSD and GDT_TS, but also the target-template sequence identity measured for the structural-alignment of the structures of the target and the template protein. Table 1 presents the AUC values for all types of search models studied here. On average, GDT_TS was the best indicator of MR success (mean AUC=0.845), RMSD was the second best (mean AUC=0.841) while sequence identity computed for structural alignment was characterized by the lowest mean AUC value, eq. 0.831. Nevertheless, according to the methodology described by Hanley and McNeil [36], these differences were not statistically significant for α=0.05. Although GDT_TS was the best indicator of MR success on average, it was not always the best predictor. For the models showed to be least useful for MR (ALL_20, BACKBONE_20, MetaMQAP-evaluated, POLYALA, POLYALA_20; see Figure 2), sequence identity for structural alignment performed better than GDT_TS.

**Table 1:** Global model quality measures in the definition of model usefulness for MR

**Predictor name**	**ALL_20**	**BACKBONE_20**	**ALL_IDEAL**	**BACKBONE_IDEAL**	**ALL_CA_IDEAL**	**BACKBONE_CA_IDEAL**	**MetaMQAP-evaluated**	**MetaMQAPclust-evaluated**	**POLYALA_20**	**POLYALA**	**MEAN (All Model Types)**
**Part A:** Area under the ROC curve *(*AUC)											
RMSD	0.842	0.846	0.845	0.838	0.841	0.831	0.804	0.841	0.864	0.856	0.841
GDT_TS	0.851	0.852	0.849	0.839	0.847	0.831	0.821	0.848	0.857	0.856	0.845
seqId_stru	0.859	0.866	0.786	0.784	0.802	0.792	0.859	0.81	0.879	0.872	0.831
seqId_seq	0.856	0.861	0.767	0.764	0.785	0.772	0.855	0.8	0.878	0.872	0.821
fraction_of_gaps	0.67	0.698	0.697	0.702	0.688	0.682	0.694	0.697	0.708	0.712	0.695
aligned_columns	0.653	0.662	0.535	0.54	0.561	0.566	0.69	0.586	0.674	0.67	0.614
global_alignment score	0.816	0.823	0.715	0.724	0.729	0.728	0.833	0.749	0.826	0.815	0.776
target length	0.672	0.674	0.545	0.556	0.579	0.577	0.701	0.61	0.693	0.687	0.629
above_1.5	0.78	0.784	0.717	0.718	0.715	0.718	0.787	0.732	0.811	0.799	0.756
abone_0.5	0.739	0.748	0.687	0.691	0.69	0.692	0.781	0.702	0.784	0.776	0.729
above_-0.5	0.686	0.695	0.621	0.623	0.633	0.645	0.756	0.635	0.694	0.697	0.669
above_-1.5	0.638	0.646	0.565	0.565	0.581	0.592	0.709	0.582	0.645	0.648	0.617
below_-1.5	0.591	0.594	0.593	0.594	0.58	0.585	0.591	0.59	0.595	0.587	0.590
AmIgoMR	0.868	0.88	0.799	0.802	0.809	0.812	0.869	0.846	0.875	0.864	0.842
**Part B:** Optimal threshold value											
RMSD	1.61	1.61	1.71	1.70	1.70	1.70	1.68	1.70	1.60	1.60	1.660
GDT_TS	79.68	79.68	78.11	77.71	78.25	78.93	79.68	78.86	79.68	79.68	79.02
seqId_stru	0.31	0.31	0.24	0.24	0.25	0.25	0.32	0.25	0.31	0.31	0.278
seqId_seq	31.05	31.05	24.02	24.02	25.07	27.06	33.05	27.06	35.05	31.05	28.84
AmIgoMR	0.29	0.29	0.18	0.17	0.20	0.20	0.29	0.19	0.39	0.29	0.249

Part A presents the accuracy of predictions of whether a model is suitable for MR. The AUC values are presented for all types of search models studied here. In addition, for each predictor also the averaged AUC value is shown. Part B shows the optimal threshold values for the predictors. Such a point reports to the value of a predictor for which the success of MR drops significantly. Predictors tested: RMSD and GDT_TS - global model quality computed by LGA program. seqId_stru and seqId_seq – sequence identity computer for structural alignment (TM-align) and sequence alignment (HHalign), respectively. The following parameters describe target-template sequence alignment made by the HHalign program: fraction_of_gaps, aligned_columns, global_alignment_score, target length, above_1.5, above_0.5, above_-0.5, above_-1.5, below_-1.5. AmIgoMR – a predictor based on the logistic regression on abovementioned HHalign parameters.

Until this point, only parameters requiring the knowledge of the native structure were tested. Thus, we decided to investigate whether it is possible to predict the probability of the success of MR using only template and target sequences, i.e. using no information about the native structure. Accordingly, we employed the HHalign program [37] to align each target sequence to sequences of the proteins that, according to DALI, are homologous to the target protein. The parameters derived from the HHalign output describe the quality of each new alignment and consequently were also tested as possible indicators of MR success. These included the following: 1) sequence identity, 2) fraction of unaligned residues, 3) global alignment score, 4) number of aligned columns, 5) target length, and 6) the column score between the query and target. As the AUC values presented in Table 1 indicate, target-template sequence identity was the best predictor of MR success (mean AUC=0.821), while the next best performing parameters were alignment score (mean AUC=0.776) and fraction of column with local alignment score above 1.5 (mean AUC=0.756).

We also searched for threshold values for the optimal predictions made by the predictors studied here (values below which the ratio of successful MR trials is expected to drop significantly). In order to estimate such a threshold value for each of the predictors studied here, a standard ROC analyzing procedure was applied, in which a threshold value was defined by the point on a ROC that was closest to the top-left corner of a ROC plot. The results presented in Table 1 shows that when GDT_TS is used to predict the MR success, the BACKBONE_20 models with GDT_TS ≥ 79.9 can be considered as useful for MR. For BACKBONE_IDEAL and MetaMQAPclust-evaluated models, the optimal thresholds of GDT_TS were 78.1 and 78.9 respectively. When sequence identity of a target-template profile alignment is used as a MR-success predictor, BACKBONE_20, BACKBONE_IDEAL and MetaMQAPclust-evaluated models were suitable for MR for the following thresholds: 31%, 24% and 27% respectively.

### Developing the method for accessing the model utility for MR

As reported above, the sequence identity between the target and the template for the search model is not as good predictor of MR success of the model as the GDT_TS, while the latter requires the knowledge of the native structure (which is not available in real life scenarios). Hence, in the second step of the study, we developed a predictor that operates only on the target and template sequences and does not require information about the native tertiary or secondary structure. Among possible indicators of MR-success the following variables were tested, obtained from the HHalign program [37]: 1) global alignment score, 2) number of aligned columns, 3) sequence identity between the target and the template, 4) fraction of unaligned residues, 5) target length, and 6-10) local alignment score (the column score between the query and target) – the number of columns aligned with a score of at least: 1.5, 0.5, -0.5, -1.5, and below -1.5. These parameters were used as a set of independent variables in logistic regression predicting the MR-success. The regression was executed in a backward mode, in which the analysis begins with a full or saturated model and independent variables are eliminated from the model in an iterative process. The accuracy of the model is tested after the elimination of each variable to ensure that the model still adequately fits the data. The analysis is complete when no more variables can be eliminated from the model. In our study, the logistic regression procedure identified eight parameters statistically important for MR-success prediction: the number of column aligned with score at least: 1.5, 0.5, -0.5, -1.5, sequence identity, fraction of unaligned residues, global alignment score, and target length. The predictor was trained and tested using a leave-one-out-cross-validation procedure, in which each learning set is created by taking all the samples except one; the sample left out serving as the test set. Thus, for n samples, the cross-validation process is repeated n times, with each of the samples used exactly once as the validation data. As depicted in Table 1, the mean AUC value for the predictor was 0.842. This shows that the predictor performs much better than sequence identity (mean AUC: 0.821) and only slightly less efficiently than GDT_TS (mean AUC: 0.845).

The abovementioned predictor can be executed via a simple web server – AmIgoMR (Am I good for MR?, http://iimcb.genesilico.pl/pawlo/amigomr/introduction.html) which, using only the sequences of the MR target and the sequence of the template, predicts whether a search with a comparative model based on a given template will find the correct MR solution***.***

We decided to conduct a test of AmIgoMR in conditions closer to real life. To do so, we downloaded the latest structures released by the PDB (October, 10^th^, 2012), which contained 147 protein entries that have been solved by X-ray crystallography. From this group, we selected 76 protein structures that were either single chain proteins or homomultimers. Among these proteins, 57 have been solved by MR. In the remaining cases phases have been obtained experimentally. We clustered members of each group at 40% sequence identity and as a result we obtained a set 31 protein structures that had been solved by MR, and 15 structures that had not so. The first group we consider as *structures proven to be solvable by MR*. MR is usually attempted as the first method before experimental determination of phases, hence the second group of protein structures that had not been solved by MR is likely to be enriched in cases *difficult for MR* – nonetheless, no information about failed MR is available for these structures.

For each of these protein structures, we queried the GeneSilico Fold Prediction metaserver [38] and then AmIgoMR. In the case of protein structures that had not been solved by MR, AmIgoMR predicted 73.3% of them as proteins for which the MR would be unsuccessful. In the case of structures proven to be solvable by MR, AmIgoMR generated 29.9% wrong predictions. Assuming the first group of proteins as negatives and the second group as positives, for the dataset of these proteins, AmIgoMR achieved sensitivity: 0.71, specificity: 0.73 and accuracy: 0.72.

### The impact of local model quality on MR success, and its dependence on global accuracy of a model

**Figure 3:**
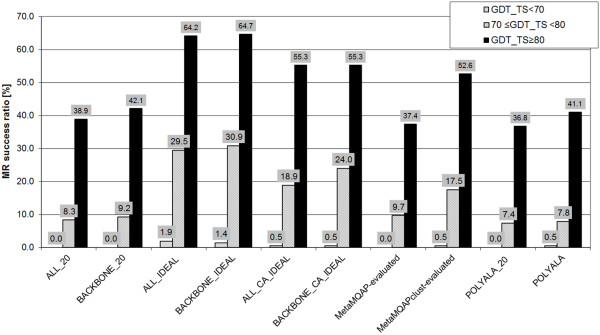
**The impact of local model quality on MR success ratio in the function of global model quality.** The success ratio is the fraction of correct MR solutions found by models of a given type. Among the model types are: 1) models with B-factor vales set to 20: ALL_20, BACKBONE_20. 2) models modified according to real accuracy of a model: ALL_IDEAL, BACKBONE_IDEAL, ALL_CA_IDEAL, BACKBONE_CA_IDEAL. 3) models modified according to predicted local quality by MQAPs: MetaMQAP-evaluated, MetaMQAPclust-evaluated; 4) templates converted into polyalanine models. The black, grey and blue bars correspond to search models of high (GDT_TS≥80), moderate (70≤GDT_TS<80) and low (GDT_TS<70) global quality.

In the previous paragraphs, we showed that the estimation of local model quality facilitated successful protein structure determination by MR. Thus, we decided to check if there is a relationship between the global quality of a model and the impact of knowledge about the local quality of the model on the MR success. Figure 3 presents MR success ratio as the function of the global accuracy of the model, expressed here by the GDT_TS score. For *search models of high quality* (GDT_TS≥80), the lowest success ratio (36.8%) was obtained by polyalanine templates with B-factor values set to 20 (ALA20 models). Utilizing comparative models, but without the information about their local model accuracy, was successful in 42.1% and 38.9% of MR searches for BACKBONE_20 and ALL_20 models respectively. Unsurprisingly, significantly higher success ratio was observed when local model quality was applied for MR. For cases, when the perfect local model accuracy was used, all-atom models (ALL_IDEAL) and backbone-atoms models (BACKBONE_IDEAL) solved 64.2% and 64.7% of MR cases, respectively. Thus, the results suggest that using the real local accuracy of a model can boost the model utility for MR by 54%.

For the *models of moderate quality* (70*≤*GDT_TS<80), we observed that real local accuracy of a model significantly enhanced the MR success ratio, compared to a standard MR searches that do not utilize any information about the local accuracy of a model. In the case of backbone-atoms models, the local quality boosted the MR success ratio by 236% (success ratio: 9.2% for BACKBONE_20 models versus 30.9% for BACKBONE_IDEAL models), and by 255% for all-atom models (success ratio: 8.3% for ALL_20 models versus 29.5% for ALL_IDEAL models).

In accordance with our expectations, the *search models of low quality* (GDT_TS<70) were practically useless for classic MR - neither ALL20 nor BACKBONE20 solved any MR cases. The same phenomenon was observed for their corresponding polyalanine templates with B-factor values set to 20. Noteworthy, the utilizing real local accuracy of a model helped to find a few correct MR solutions with models of quality below GDT_TS 70. In our study, all-atom models combined with their local model quality were successful in 1.9%.

In the preceding paragraph, we demonstrated that the real local accuracy of a model had a significant positive impact on the model usefulness for MR. Compared to the MR searches using polyalanine templates with B-factor values set to 20 (POLYALA_20), utilizing local accuracy of a model (as it was shown for BACKBONE_IDEAL models) can increase the MR success ratio by 75% for models with GDT_TS ≥80 and by 317% for models of lower global accuracy (70*≤*GDT_TS<80).

According to our data, local model quality was critical for solving MR cases with the use of low quality models (GDT_TS<70). For such cases we achieved a 1.4% success ratio using BACKBONE_IDEAL models, while for classic MR (POLYALA_20) the ratio was 0%.

### The utility of model quality prediction by MetaMQAP and MetaMQPAcons in MR

In the previous paragraphs, we have shown that the real local accuracy of a model positively influences MR searches. Nevertheless, the real quality remains unknown until the final structure of a protein is solved. However, there has recently been a tremendous development in the field of methods that predict local model quality without any knowledge of the native structure. Since there are two main classes of Model Quality Assessment Programs (MQAPs): “true MQAPs” and “clustering MQAPs”, we decided to verify whether the MQAPs of either type are able to enhance the model utility for MR. To achieve that, we tested two MQAPs developed in our laboratory: MetaMQAP (a “true MQAP”) and MetaMQAPclust (a “clustering MQAP”). Thus, we created two groups of search models, the first group (MetaMQAP-evaluated) containing models with their B-factor values set according to the deviation predicted by MetaMQAP. Since MetaMQAP predicts only the deviation of the C-α atom for a given residue, the B-factor values of the remaining atoms were set to the B-factor value of the C-α atom. Moreover, all side-chain atoms were removed from the models. The same procedure was applied to produce the second group of models (MetaMQAPclust-evaluated), but here we used the MetaMQAPclus method to predict the model accuracy.

According to the results presented in Figure 2, the MetaMQAPclust-evaluated models helped to solve 22.6% of MR cases, showing that using MetaMQAPclust enhanced the success ratio by 38,7% compared to the BACKBONE_20 models. No improvement over unmodified models was observed in the case of the MetaMQAP-evaluated models.

Models with GDT_TS≥80, with B-factor values changed according to the MetaMQAPclust prediction, solved 52.6% of MR cases. Thus, for those models the MR success ratio is improved by 25% compared to BACKBONE_20 models. For the lower-quality models (70≤GDT_TS<80), MetaMQAPclust predition enhanced the MR success ratio by 90% compared to BACKBONE_20 models. Finally, MetaMQAPclust helped in obtaining the correct MR solution with a model having GDT_TS score of 69.2, i.e. demonstrated that the use of MQAPs can push the threshold of applicability of MR for models with GDT_TS < 70.

## Discussion

We have shown that using local model quality to recalculate the B-factors of a search model increases the success ratio of MR. For our dataset of 615 search models, the real local accuracy of a model increases the MR success ratio by 101% compared to corresponding polyalanine templates. On the contrary, when local model quality is not utilized in MR, the computational models solved only 4.5% more MR searches than polyalanine templates. This suggests that comparative models can be very useful in MR, if their local accuracy is taken into account.

The presented ROC analysis (Table 1) points to the fact that the GDT_TS between the model and the native structure is a better measure for assessing model utility for MR than RMSD or sequence identity computed for structure-based target-template alignment. The findings are consistent with those of Giorgetti and Tramontano [10]. They also reported that GDT_TS >84 was sufficient to guarantee success in MR and that models with GDT_TS <80 were not successful in MR. In our study, we showed that MR can be solved even using models with GDT_TS<80. This inconsistency may be explained partially by the fact that in our MR workflow two MR programs: AmoRe and MOLREP, were used independently, thus increasing the likelihood of determining the correct MR solution. In contrast, the Giorgetti’s MR workflow [10] used only MOLREP to find the initial solution, and then AmoRe just for the refinement of an initial MR solution. Moreover, the search models studied here were trimmed by removing insertions longer than 8 residues. We decided to do that because insertions are more likely to contain significant errors, and if they are not removed, the errors may cause a model to fail in MR [8,39].

An important finding comes from the comparison between MR procedures that use/do not use the local accuracy of a model. For the MR searches not utilizing such accuracy the success ratio drops significantly for GDT_TS ≤ 79.7 (BACKBONE_20). When local accuracy of a model is used, the MR success ratio declines significantly for GDT_TS ≤ 77.7 (BACKBONE_IDEAL). Interestingly, a marginal 2-unit change in GDT_TS score is accompanied by an over 2-fold change of the observed MR success ratio (MR success ratio: 31.4% and 16.3% for BACKBONE_IDEAL and BACKBONE_20 respectively). A possible explanation for this might be that to solve a phase problem by MR a particular fraction of well-modeled atoms (with a deviation <1.5Å in the case of MR using the resolution limit of 3Å; see Additional file 1: Figure S3) is always required by a MR algorithm. If this criterion is satisfied, the likelihood of MR’s success can then be significantly boosted by making incorrectly modeled atoms (those with deviation ≥1.5Å) less important for the calculation of structure factors. In this paper, we have shown that this can be achieved by setting temperature factor values according to the local accuracy of a model, and then solving the phase problem by using MR programs that take into account these B-factor values (e.g. AMORE and MOLREP).

We observed that the knowledge of the local quality of a search model is crucial for MR searches with models of moderate quality (70≤GDT_TS<80). Backbone variants of models solved only 20 phasing attempts (9% of 217 models). In contrast, the same models found correct solution for 67 cases (31% from 217), provided they had their B-factors values modified according to the real local accuracy of a model. The results proved that the real local quality of a model increases the model usefulness by 235%. In comparison, for the highest quality models (GDT_TS ≥ 80), backbone models were successful in 80 cases (42% of 190 models) of MR searches, while a combination of the backbone models with their real local accuracy found the correct solution in 105 cases (66% of 190), thus increasing the MR success by 54%. The difference suggests that moderate quality models still contain a required fraction of well-modeled atoms (deviation <1.5Å), but that to make these models significantly more useful for MR, the influence of local errors on the calculating of structure factor must be reduced.

Not only have we evaluated the potential of using the perfect local model quality for MR but also tested whether the use of Model Quality Assessment Programs (MQAPs) can increase the MR success ratio. Between the two tested MQAPs: MetaMQAP (a trueMQAP) and MetaMQAPclust (a clustering MQAP), only the MetaMQAPclust method has been shown to significantly boost the MR success ratio. For our dataset of 615 search models, a workflow combining MR with the MetaMQAPclust prediction found 139 correct solutions, which is 45% more compared to polyalanine models (96 solved cases, POLYLALA) and 39% more compared with backbone-atoms models with B-factor values set to 20 (BACKBONE_20). The observation shows that current MQAPs can significantly increase the MR success ratio.

Since MetaMQAPclust predicts only the accuracy of C-α atoms, it is very important to stress that the upper limit of the usefulness of the MQAP for MR is defined by the above-mentioned results for BACKBONE_CA_IDEAL models. In these models, for each residue, the real deviation of a C-α atom was used to modify the B-factor values of remaining atoms. We showed that BACKBONE_CA_IDEAL solved 158 MR searches. This means that an ideal C-α-MQAP would make MR find 65% more solutions than polyalanine-based MR, and only 14% more than MR that uses the current MetaMQAPclust program. This shows that further development of MQAPs predicting the accuracy of C-α atoms is still needed. However, MR using both all-atom models and real local accuracy of these atoms - (ALL_IDEAL models) - solved 190 MR cases, which is 37% more that MetaMQAPclust-based MR. Thus, we postulate that MR would benefit most from an MQAP capable of predicting local model quality (in Angstroms) for *all atoms* in a model. To our best knowledge, such a method has not been developed so far.

Our study is admittedly biased towards an optimistic view of the possibilities of combining comparative models and local model quality with the MR technique. The main limitation lies in the fact that the comparative models tested here were created on the basis of structural target-template alignments, thus producing more accurate alignments than sequence- or profile-based alignment methods. We decided to use structural alignments instead of sequence- or profile-based ones for the following reasons: First, there are plenty of different sequence- or profile-based methods and selecting one or a few of them would also bias our results towards these method(s). Second, there has been a continuous development in sequence alignment methods based on profile-profile comparison, thus the accuracy gap between structural and non-structural alignments methods has decreased. Finally, as shown by the Godzik group [8], the likelihood of solving the phase problem for a given protein increases for extensive MR searches using the population of alternative alignments. Therefore, the structural alignment can be considered as a representative of these alternative alignments. Considering all these assumptions, we are sure that not only does our study present the theoretical upper limitations of combining local model accuracy with MR, but also with the continuous development of profile-profile alignments methods it offers a new practical approach for MR.

## Conclusions

In conclusion, we have shown that using comparative models only marginally increases the MR success ratio in comparison to the polyalanine structures of templates. However, the situation changes dramatically once comparative models are used together with their local accuracy. We found that the lower the global quality of a model, the more significant is the impact of knowledge about the local quality of the model on the utility of the model for MR. We demonstrated that the use of a local accuracy of a model greatly increases the likelihood of successful MR calculations for search models with GDT_TS ≤ 80. We proved that the real local accuracy of a model enhances the MR success ratio threefold, for polyalanine models with GDT_TS between 70 and 80. Surprisingly, the perfect local accuracy of a model can make MR find correct solution even with a model having GDT_TS ≤ 70. Our MR workflow was successful with a model that had GDT_TS=67.2 and target-template sequence identity of 16%.

Since the real local accuracy of a model is not known once the structure of a protein is solved, we tested whether Model Quality Assessment Programs (MQAPs) can improve the utility of theoretical models in MR. Between the two programs tested in the study, MetaMQAP (true MQAP) and MetaMQAPclust (clustering MQAP), only the MetaMQAPclust significantly boosted the MR searches. This suggests that current clustering MQAPs can increase the model utility for MR. In addition, in our opinion, there is a great need for the development of MQAPs capable of predicting the deviation for all atoms in a model, not only C-α ones.

Inspired by these findings, we have added a new functionality to GeneSilico Fold Prediction Metaserver [38] that will allow building models that are more useful for MR searches. Thus, for a submitted target sequence, the GeneSilico metaserver generates many alternative models, then these models are trimmed in order to remove insertions longer than 8 aa, next MetaMQAPclust is executed to assess the local quality of these models. Finally, these model’s B-factor values are modified according to MetaMQAPclust prediction. Such models can be easily downloaded to the user’s computer, and then used as an input for MR searches (see Additional file 1: Figure S1).

Additionally, we have developed a simple method, AmIgoMR, to predict if MR search with the template-based model will find the correct solution. In our MR-workflow, AmIgoMR predicts the MR success almost as accurately as GDT_TS does. Nevertheless, for different home-made MR workflows, AmIgoMR may less accurately estimate the absolute probability of solving MR with a given model. However, in such cases our method can still help to rank models according to their usefulness for MR. The AmIgoMR program can be executed as a standalone web application: http://iimcb.genesilico.pl/pawlo/amigomr/introduction.html and is also automatically run for predictions returned by the GeneSilico metaserver.

## Competing interests

The authors declare that they have no competing interests.

## Authors’ contributions

MP and JMB conceived of the project, MP developed the MR-pipeline and generated data presented in the publication. MP analyzed the results, drafted the manuscript and prepared the figures. JMB edited the manuscript. Both authors analyzed and interpreted the data. All authors read and approved the final manuscript.

## Supplementary Material

Additional file 1 **Supporting Information. Table S1:** Basic statistics of models constructed for all target proteins. **Figure S1:** The results page of GeneSilico Fold Prediction Metaserver and two its new functionalities. **Figure S2:** Rebuilt of models in our MR workflow. **Figure S3:** Correlation between the value of atom deviation and MR success. Click here for file
