# Supplementary material for: The utility of comparative models and the local model quality for protein crystal structure determination by Molecular Replacement
Source: BMC Bioinformatics. 2012 Nov 5;13:289. doi: 10.1186/1471-2105-13-289 (PMC3534383; doi:10.1186/1471-2105-13-289)

# Supporting Information

**The utility of comparative models and their local quality for protein crystal structure determination by Molecular Replacement**

Marcin Pawlowski1, Janusz M Bujnicki1,2*^1^*

*Laboratory of Bioinformatics and Protein Engineering, International Institute of Molecular and Cell Biology, ul. Ks. Trojdena 4, PL-02-109 Warsaw, Poland*

*^2^Bioinformatics Laboratory, Institute of Molecular Biology and Biotechnology, Faculty of Biology, Adam Mickiewicz University, ul. Umultowska 98, PL-61-614 Poznan, Poland*

# Tables

## Table S1. Basic statistics of models constructed for all target proteins.

The table presents some parameters calculated for the dataset of 615 models. To measure the global accuracy of these models cRMSD and GDT_TS are used. For these parameters minimum, maximum and mean values are presented.

| PDB code | Target protein resolution [Å] | Number of comparative models | cRMSD [Å] | | | GDT_TS | | |
| --- | --- | --- | --- | --- | --- | --- | --- | --- |
|  |  |  | min | max | mean | min | max | mean |
| 1o0x | 1.90 | 22 | 0.14 | 2.06 | 1.52 | 67.56 | 100.00 | 79.23 |
| 1qy6 | 1.90 | 76 | 0.18 | 2.21 | 1.90 | 46.63 | 100.00 | 66.51 |
| 1vlc | 1.90 | 71 | 0.16 | 2.53 | 1.84 | 50.77 | 100.00 | 73.26 |
| 1wgb | 2.00 | 14 | 0.14 | 1.78 | 1.51 | 75.64 | 100.00 | 79.71 |
| 2gw2 | 1.80 | 48 | 0.55 | 1.80 | 1.03 | 72.19 | 98.84 | 92.72 |
| 2gzv | 1.12 | 86 | 1.21 | 2.40 | 1.72 | 53.02 | 90.58 | 77.79 |
| 2h58 | 1.85 | 77 | 1.31 | 2.70 | 1.94 | 31.89 | 86.17 | 58.01 |
| 2he4 | 1.45 | 87 | 1.03 | 2.49 | 1.73 | 60.56 | 90.73 | 77.39 |
| 2hsb | 1.95 | 3 | 1.54 | 1.75 | 1.66 | 79.61 | 82.20 | 80.84 |
| 2ict | 1.63 | 42 | 1.58 | 2.83 | 1.98 | 42.86 | 83.61 | 61.21 |
| 2iwn | 1.35 | 89 | 1.19 | 2.41 | 1.86 | 55.91 | 88.61 | 74.44 |
| **overall** | **1.62** | **615** | **0.14** | **2.83** | **1.76** | 31.89 | **100.0** | **73.00** |

**Figures**

**Figure S1. The results page of GeneSilico Fold Prediction Metaserver and two its new functionalities.**

To submit a target sequence to GeneSilico Fold Prediction Metaserver, one needs to follow the link: <https://genesilico.pl/meta2/prediction>. Once the user has got the result for the submitted target sequence, GeneSilico Fold Prediction Metaserver offers two new functionalities:

1)“Execute AmiIGoMR” collects the target sequence and the pdb codes of all the proteins found by GeneSilico Metaserver to be likely structurally similar to the target protein. Then AmIGoMR is executed in order to predict the probability of Molecular Replacement (MR) success when a comparative model created on the base of a given template is used.

2)“*Generate models for Molecular Replacement (MR)*” builds models that are more useful for MR searches. First, GeneSilico Fold Prediction Metaserver generates many alternative models for the target protein, then insertions longer than 8 aa are removed from these models. Next, MetaMQAPclust is executed to assess the local quality of these models. Finally, these model’s B-factor values are modified according to MetaMQAPclust prediction, and the user is send an email with a link to download these models.


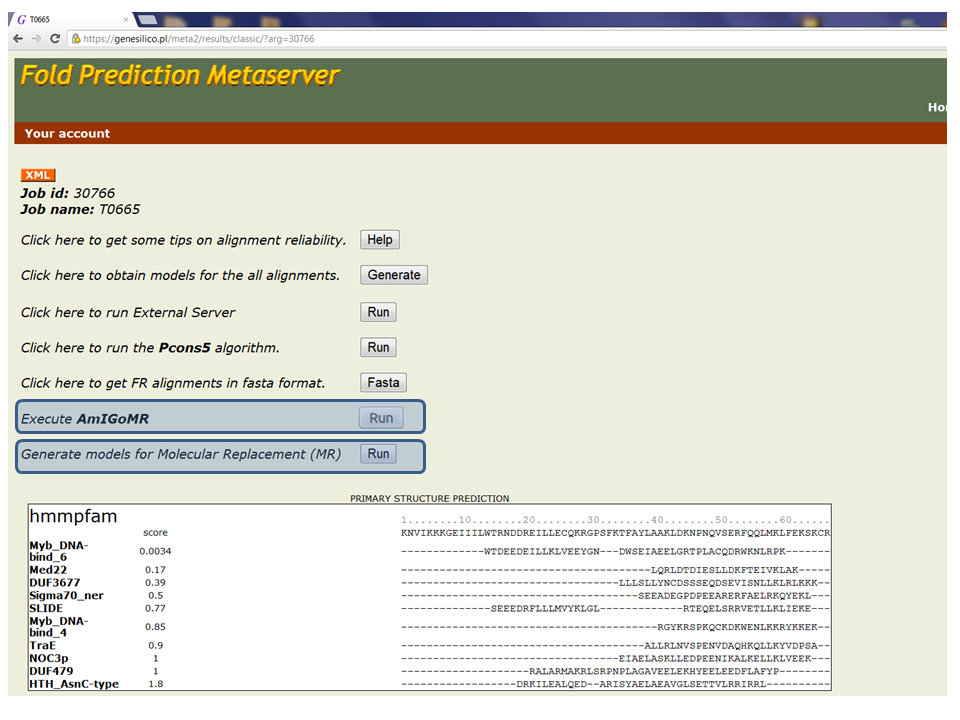


**Figure S2. Rebuilt of models in our MR workflow.**

The chart presents the histograms of the fraction of rebuild C-alpha atoms for BACKBONE_IDEAL models and POLYALA_20 models. The fraction is set to 0 for MR cases where no model was created.

**
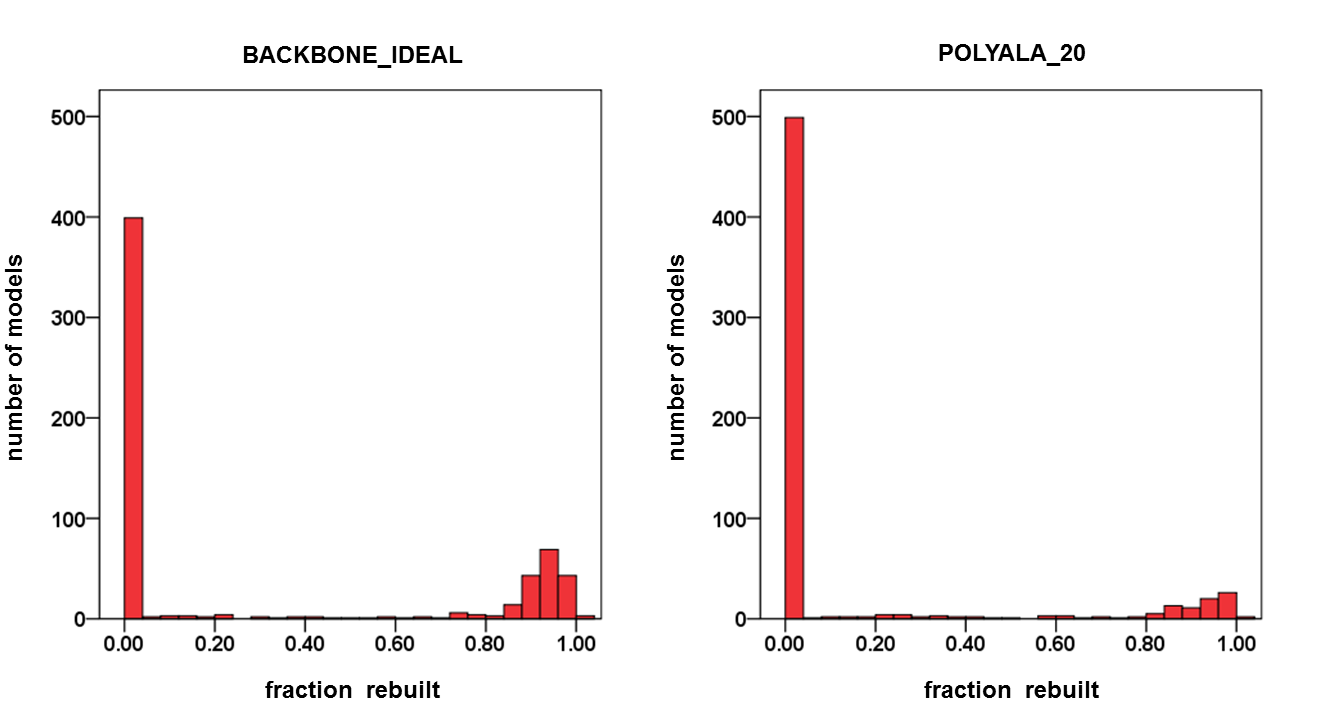
**

**Figure S3. Correlation between the value of atom deviation and MR success.**

The chart presents correlation coefficient between fraction of C-α atoms characterized by a given error in a model and the model usefulness for MR. Deviations in the range of 0- 0.5Å are marked as 0.5, deviation in range of 0.5-1.0Å as 1, etc. The analysis is made for BACKBONE_IDEAL and POLYALA_20 models – the most and the least useful models for MR, respectively. In addition, also the averaged correlation coefficients over all model types is presented - OVERALL. In general, once the fraction of C-alpha atoms that are characterized by an error ≤1.5Å increases in a model, this model usefulness for MR also increases. The reverse relationship is observed for residues with en error bigger than 1.5Å. Since the resolution limit of 3Å was used in MR searches, the atoms with deviation at least 1.5Å, which is a half of the wave-length, are shown to have a negative impact on MR success.


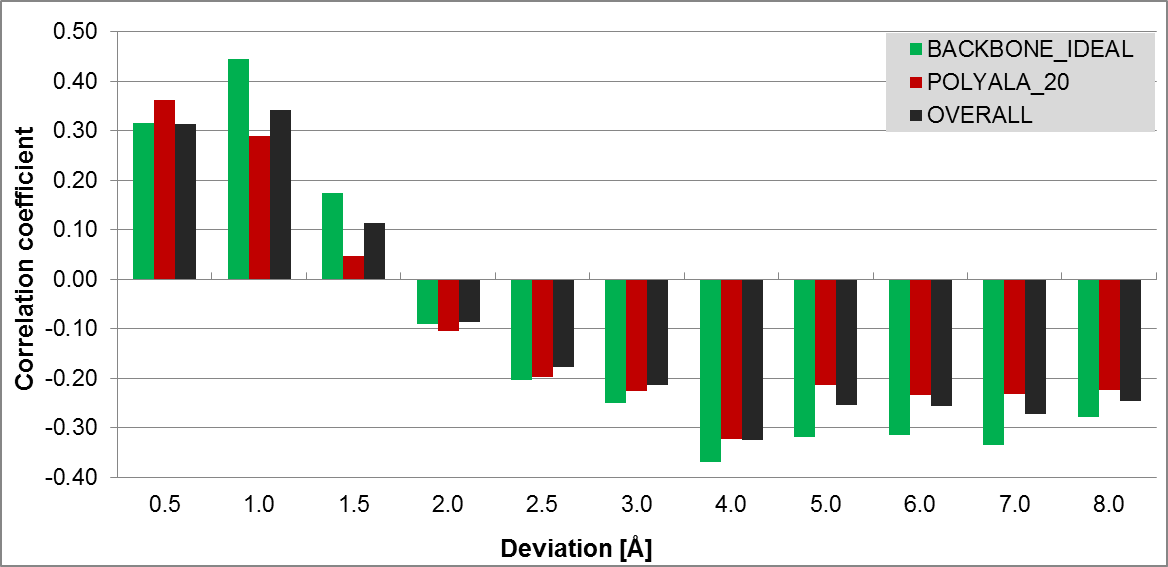

Supplement: Additional file 1 — Supporting Information. Table S1: Basic statistics of models constructed for all target proteins. Figure S1: The results page of GeneSilico Fold Prediction Metaserver and two its new functionalities. Figure S2: Rebuilt of models in our MR workflow. Figure S3: Correlation between the value of atom deviation and MR success. [file 1471-2105-13-289-S1.docx]
